# A Method for the Automated, Reliable Retrieval of Publication-Citation Records

**DOI:** 10.1371/journal.pone.0012133

**Published:** 2010-08-19

**Authors:** Derek Ruths, Faiyaz Al Zamal

**Affiliations:** Department of Computer Science, McGill University, Montreal, Quebec, Canada; Aarhus University, Denmark

## Abstract

**Background:**

Publication records and citation indices often are used to evaluate academic performance. For this reason, obtaining or computing them accurately is important. This can be difficult, largely due to a lack of complete knowledge of an individual's publication list and/or lack of time available to manually obtain or construct the publication-citation record. While online publication search engines have somewhat addressed these problems, using raw search results can yield inaccurate estimates of publication-citation records and citation indices.

**Methodology:**

In this paper, we present a new, automated method that produces estimates of an individual's publication-citation record from an individual's name and a set of domain-specific vocabulary that may occur in the individual's publication titles. Because this vocabulary can be harvested directly from a research web page or online (partial) publication list, our method delivers an easy way to obtain estimates of a publication-citation record and the relevant citation indices. Our method works by applying a series of stringent name and content filters to the raw publication search results returned by an online publication search engine. In this paper, our method is run using Google Scholar, but the underlying filters can be easily applied to any existing publication search engine. When compared against a manually constructed data set of individuals and their publication-citation records, our method provides significant improvements over raw search results. The estimated publication-citation records returned by our method have an average sensitivity of 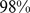 and specificity of 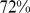 (in contrast to raw search result specificity of less than 10%). When citation indices are computed using these records, the estimated indices are within 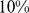 of the true value, compared to raw search results which have overestimates of, on average, 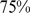.

**Conclusions:**

These results confirm that our method provides significantly improved estimates over raw search results, and these can either be used directly for large-scale (departmental or university) analysis or further refined manually to quickly give accurate publication-citation records.

## Introduction

It is commonplace for an academician's publication-citation record, the list of her publications as well as citation counts, to be used as a proxy for her professional output. Popular measures such as the h- and e-indices ([1], [2]) distill such lists of publications and citation counts into scores which can heavily influence faculty recruitment, tenure recommendations, as well as department and university performance indicators. As we will show, inaccurate publication-citation records can lead to dramatic inaccuracies in indices and scores derived from them. Because of the prevalent use of such scores in important matters, obtaining accurate publication-citation records can be an endeavor with far-reaching implications.

In this paper, we exclusively consider the problem of large-scale publication-citation record (hereafter, *PCR*) reconstruction and analysis—as is often done in the case of cross-departmental performance comparisons (as was the circumstance which motivated our interest in this research topic), school-wide impact studies, and other investigations which involve the acquisition of large numbers of PCRs. In these cases, the person building a specific individual's publication-citation record will rarely be the owner of the PCR itself. This fact complicates the task of obtaining an accurate PCR in several ways. If we make the generous assumption that the subject has provided an accurate list of publications, the first challenge encountered is simply the tedious and time-consuming task of collecting citation numbers from Google Scholar [3], Web of Science [4], or myriad other services. In reality, the subject's complete and accurate PCR generally will only be available to tenure committees and prospective employers. Those conducting larger-scale PCR analysis will often find themselves hampered by researchers' webpages that provide incomplete, poorly-formatted, or all-together missing publication lists. In fact, even were all publication information available, given the number of individual PCRs involved in comparing several departments (e.g., over 200 individuals in the case of our study), sufficient time is often not available to build each manually.

These complications have resulted in a general dependence on online publication search engines such as Google Scholar, Web of Science, and others which provide the ability to quickly search for publications (and their citation counts) authored by specific individuals identified by name. Names, however, are often not unique identifiers. As we establish in later sections, this name ambiguity causes search engines to return publication records that are orders of magnitude larger than the true PCR for the individuals of interest. Citation indices computed using such records can be overinflated on similar scales.

With this situation in mind, we pose the *Publication-citation Record Estimation Problem*: given an individual of interest, we seek an accurate estimate of the individual's publication-citation record using only available information about the individual, search engines, but stopping short of manually processing the individual's publication list (should such information exist).

In this project, we present a computational method that uses contextual information about the individual of interest in order to obtain an accurate estimate of her publication-citation record. The method works by applying a series of four filters to the results returned by an online publication search engine. The first filter ensures publications fall within the timespan of the individual's career. The second applies a stringent name-matching criterion to each candidate publication's author list. The third filter, which we consider to be the core conceptual contribution of this work, uses vocabulary from the individual's research and/or publication webpage to identify publications that belong in the individual's field of research. The final filter removes any duplicates present in the candidate publication pool.

From the outset, we stress that our method's contribution is to remove publications from search engine results that most likely do not belong to the author specified in the query. Our approach does not address any issues with limited journal coverage or inaccurate citation counts: these are limitations of the search engine. To our knowledge, all approaches, whether manual or automated, are limited in this regard. However, as publication search engine accuracy and coverage improves, so too will the results of our method.

To assess our method's performance, we manually constructed the PCRs for 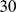 faculty members selected at random from all wide array of scientific disciplines and academic institutions. When given the names and webpage publication-lists for these individuals, the estimate PCRs returned by Topp had an average sensitivity of 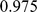 and specificity of 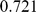. This demonstrated a marked improvement over the unfiltered search results which had a specificity of 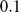. Errors in the h- and e-indices computed using the estimated PCRs were off by approximately 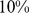
—one tenth that of the raw unfiltered results. It is worth noting that some of the inaccuracies present in the estimates returned by our method could be traced back to misspellings and other low-level errors in the search engine results. This suggests that as publication search engines improve, so will the estimates returned by our method.

A final contribution of this paper is the use of our methods to consider the effect that errors in PCR estimates can have on citation indices: showing that the raw results of publication search engines can overestimate citation indices by more than a factor of two.

To our knowledge, ours is the first method which introduces this degree of sophistication to the PCR estimation problem. Various websites such as the *HView Visualizer* ([5]) and *scHolar index* ([6]) as well as software applications such as *Publish or Perish* ([7]) provide convenient interfaces for querying and viewing the results of Google Scholar, a popular publication search engine. These tools, however, all rely on name uniqueness (applying no additional filters or processing to the name results) and, therefore, have accuracies which are comparable with unfiltered publication searches by author name.

In the following sections, we first describe our method and the different filters used to select the final estimated publication-citation record. We then evaluate the performance of our method and the various filters that comprise it on a manually curated data set of PCRs. We compare our method's accuracy to unfiltered results returned by search engines. Finally, we consider the impact that error in publication-citation record estimates can have on various citation indices.

## Methods

**Figure 1:**
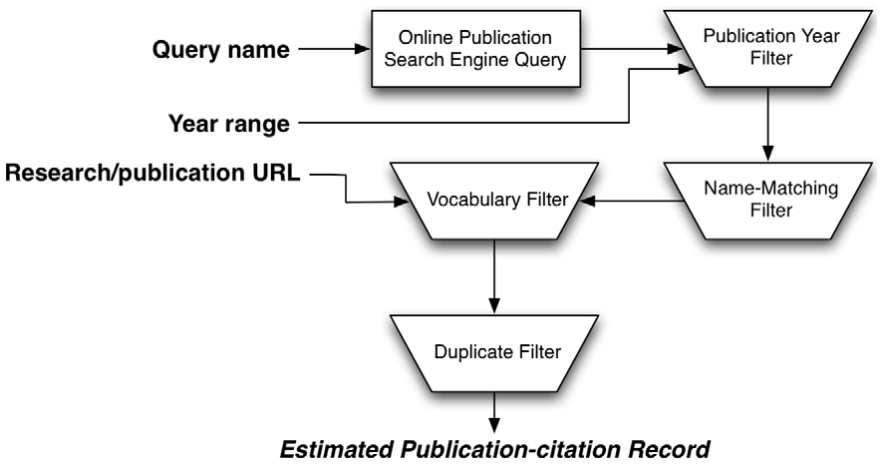
The functional components involved in a publication-citation record query performed by our method. Input for the query is the name of the individual (*Query name*), the range of years during which the individual was publishing (*Year range*), and a URL to a webpage that contains either a list of the individual's publications or a description of the individual's research (*Research/publication URL*). The Topp engine then applies four filters. The result is the set of publications that passed all the filters.

Figure 1 depicts the conceptual pieces that, together, comprise the method. The method takes three pieces of input: the name of the individual whose publication-citation record is being constructed, the year range during which that person published works, and a URL containing either a list of the individual's publications or a rather comprehensive description of the individual's research. The output of the method is a list of publications (with citation counts where available). If indicated, the method also returns various citation measures such as the h- and e-indices.

To explain our method, we use the following terms:

- q.lname - the last name of the query individual
- q.fname - the first name of the query individual
- q.finitial - the first initial of the query individual
- q.minitials - the initials of the query middle names, all concatenated in one string (e.g., for ‘Bob John Paul Smith’, q.minitials = ‘JP’)
- q.initials - the initials of the query individual's first and middle names, all concatenated in one string (e.g., for ‘Alan M Turing’, q.initials = ‘AM’)
- q.syear - the start year of the query individual's publication career
- q.eyear - the end year of the query individual's publication career
- q.url - a URL to the individual's publication or research webpage
- p.initials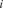 - the initials of publication 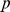
's 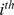 author's first and middle names, all concatenated in one string
- p.lname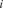 - the last name of publication 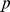
's 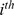 author's last name
- p.title - the title of the publication
- p.year - the year of the publication was published

Note that the individual's name, URL, and year range are inputs for our method. All query initials can be derived from a full name given to the method, thus only q.fname,q.minitials,q.lname,q.syear,q.eyear,and q.url are specifically required for the method to run.

We discuss our method by first describing how existing online publication search engines return results—a matter that provided important motivation and design constraints for our method. We then discuss the way in which a query is made, individual filters applied, and the ordering of filters used.

### Publication Search Engine Queries

The objective of our method is to identify the publications authored by a specific individual. Most online search engines support the advanced search feature of looking for publications authored by someone with a specific name. As an example, in the case of Google Scholar, this is done by using the query syntax ‘author:“
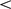
query string
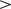
”’.

While the internals of most popular publication search engines are not public knowledge, running several queries quickly reveals that when such a search is performed, the backend algorithm selects publications by applying a lenient filter to author names. While we were not able to formalize exact rules, the behavior of the algorithm appears to depend on the structure of the input:

1. author:“q.initials q.lname” - return all publications such that for each publication 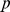, 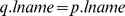 and 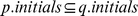, where 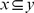 is true if the characters of 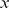 are a subset of the characters in 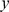.
2. author:“q.fname q.lname” - return all publications such that for each publication 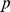, 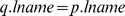 and either 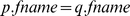 if 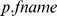 is known or 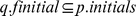 if 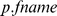 is not known.
3. author:“q.fname q.minitials q.lname” - return all publications such that for each publication 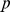, 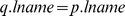 and either 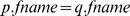 if 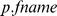 is known or 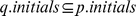 if 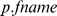 is not known.

The behaviors above are little more than guides to how queries will generally perform: a quick survey of search query results identifies many results that break the rules formalized above. However, the rules above, such as they are, are given in order of increasing stringency: in general search queries of type 1 will return more results than those with 3. The more common an individual's name, the more stringent the rule used must be—this is our general observation and recommendation, but does not guarantee improved results. Overall, these details serve to illustrate that slightly modifying how the query individual's name is used to construct the query can have a significant impact on the initial set of candidate publications returned by the search engine. Our method supports all of the different query structures discussed above, although at present the structure used must be manually selected when running the query. The default choice is author:“q.fname q.minitials q.lname”. Except where noted, all results used in this paper were generated using this default setting.

### Publication filters

As shown in Figure 1, our method consists of four filters that remove publications which do not pass specific criteria. The final estimate publication-citation record returned by our method is the set of publications, initially returned by the search engine query, which pass *all* of the filters. Formally,
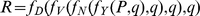
where 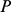 is the set of publications returned by the online search engine, 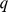 is the query information, and 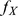 is one of the filters described next. We now discuss the motivation for each filter as well as the way in which each is designed and implemented.

#### Filter 1: publication year

Particularly for individuals who have only recently developed a citation record, the publication year can rapidly cull unrelated publications from the set of candidates. This filter, the simplest of the four used in our method, takes a start and end year and selects only publications whose publication date falls within the range:
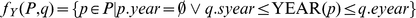
where 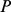 is the set of input publications for the filter and 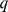 is the query information. Note that the condition for selection also admits publications with no year, since online publication search engines can occasionally omit publication years.

#### Filter 2: name-matching

The search engine rules discussed earlier suggest that under many circumstances search engines will return publications with an author whose initials matches *any part* of the query name's initials. Therefore, the query author:“D Ruths” will match publications containing authors with names such as: *D Ruths*, *DE Ruths*, *AD Ruths*, etc… This last name, *AD Ruths*, is irrelevant to our individual of interest, however, because we can assert that “D” is the first initial of the author's name. The name-matching filter applies a generalized form of this logic to all publications. Formally,
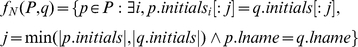
where 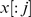 is the 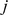-length prefix of string 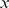. Effectively, this ensures that both the 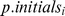 and 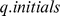 agree on the shorter of the two strings, regardless of whether the publication or query has more initials specified.

#### Filter 3: vocabulary agreement

As mentioned in the introduction, name uniqueness is not a sufficiently rigorous criterion to discern the publications that belong to a given individual: many individuals share the same name, many more share the same last name and first and middle initials. In light of this fact, we require another criterion to decide which papers belonging to two or more individuals with the same name were actually authored by the individual of interest. A central insight we present in this paper is that of using domain-specific vocabulary to recognize papers belonging to a specific individual. Particularly in science and engineering, the titles of papers contain a significant amount of domain-specific vocabulary which will not appear in any other domain (e.g., names of genes, chemical compounds, and design patterns). We use an individual's online research statement or publication webpage to define the set of domain-specific terms which will be used. Any candidate publication containing a sufficient number of these terms will be accepted as belonging to the individual's research area and, therefore, to the individual of interest. It is important to note that this makes the strong assumption that two people who work in areas which share the same domain-specific vocabulary and share the same name are highly unlikely. We acknowledge that this is a potential source of error in our method, though in our validation and in subsequent use of the tool, we have not observed this to be a significant source of error even for very common names. Nonetheless, we identify this issue for consideration in future work.

In order to implement the logic described above, the vocabulary filter works as follows:
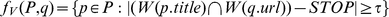
where 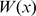 returns the words in the string 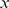 (in the case of 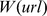, the function returns all alphanumeric words in the webpage referred to by the URL), and 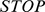 is a set of stop words. Typically, stop words are words that are necessary to the construction of a valid sentence (e.g., ‘the’,‘and’,‘with’); we expanded the stop words to include vocabulary that is highly shared among scientific disciplines (e.g., ‘analysis’ and ‘theory’) in order to avoid detecting similarity due to general scientific language. 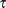 is a threshold that determines the minimum number of domain-specific terms a title must contain in order to pass the filter. We have found 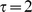 to be the best threshold, and in the next section, we examine the effect of different values of 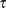 on the performance of this filter to substantiate this claim.

#### Filter 4: duplicate removal

Because some online publication search engines index multiple publication sources whose coverage of publications may overlap, it is not uncommon for a search query to return several records that correspond to the same publication. Unfiltered, each of these duplicate records increases the publication and citation counts: when many publications are involved the effects of duplications can be substantial. A complicating issue is that it is also common for individuals to have different publications that share identical or very similar titles. For this reason, we designed the duplicate filter to be very lenient, accepting any publications in which authors or publications years differ (even though occasionally these details may be reported differently for the same publication by different sources). The resulting filter takes the form:
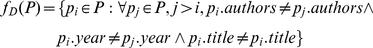
where 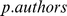 is a canonical version of the author string (i.e., initials lname, initials lname,…).

### Ordering of filters

The order in which the filters are applied (see Figure 1) was influenced by considering the extent to which specific orderings influenced correctness and performance.

#### Ordering and correctness

The year, name, and vocabulary filters all operate on publications independently, rendering their contribution to the filtering step order-independent. The behavior of the duplication filter, however, can be influenced by the set of candidate publications present. The idea behind the duplication filter is to take two publications that definitely belong to the same individual and remove one of them to avoid double-counting. We have highest certainty that two publications belong to the individual of interest after all other filters have been applied. Thus, the duplication filter should be applied last.

#### Ordering and performance

Given that the year, name, and vocabulary filters could be arranged in any relative order, they were placed in order of increasing computational complexity: the year filter requires a simple linear-time walk through the publications with a simple numerical comparison at each step; the name filter requires a slightly more involved string comparison; and the vocabulary filter is the most expensive due to the set intersections that must be done. This said, given the scale of the publication list and word sets being considered, performance was not observed to be a significant issue. It is likely that for many inputs, any ordering of these filters would yield indistinguishable performance.

## Results and Discussion

There are two main themes to the experimental studies we conducted in this project. First, we evaluated the accuracy of our tool, both against a manually curated data set as well as against the results of existing tools. We then used our method to study the effect of overestimation of publication-citation records on the accuracy of citation indices—an important issue to understand in light of the wide-spread use of such measures to evaluate individual, department, and university performance.

Initially, we considered using our method with three different publication search engines: Google Scholar, PubMed, and Web of Science [3], [4], [8]. PubMed does not provide citation information and, therefore, was dropped. The Web of Science search engine does not index as wide an array of journals as Google Scholar - in experiments, we found that queries run against Google Scholar consistently outperformed Web of Science queries in publication coverage. Therefore, results in this section were generated using Google Scholar [3]. Besides the notable performance comparison, Google Scholar is an entirely free web-service which makes it accessible for anyone to use. It is worth noting that, while the prototype implementation of our method is built to interface with Google Scholar, the internals of our method are entirely separated from the choice of search engine.

### The dataset

In all of the analysis that follows, we used a manually curated data set of 30 faculty members selected from random international institutions and random scientific fields. Their publication records were built from publication lists linked to their webpages. While it is possible that some of these lists are incomplete, we minimized this risk by only including individuals whose publication lists were indicated to be complete. In an attempt to eliminate any risk of selection bias in our testing and results, this data set was collected prior to performing any tests of the method and no changes were made to the membership of this dataset based on performance, availability of individual information, or any other considerations.

### Method validation

We investigated three different aspects of our method's performance: the contributions that the different filters make to its accuracy, the influence of input domain-specific vocabulary (the content of the URL) on accuracy, and the effect of different values of 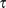, the vocabulary overlap size, on accuracy.

### Computing sensitivity and specificity

Throughout this method validation section, we evaluated accuracy in terms of sensitivity (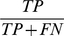) and specificity (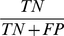). For a given individual, we have the candidate publications returned by the search engine, 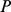, the estimated publication-citation record returned by our method, 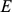, and the manually curated publication record, 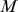. Given these results, we define the following:

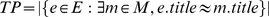
,

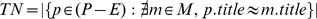
,

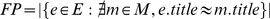
, and

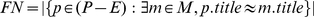
,

where 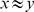 iff 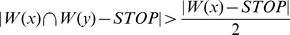. In theory, the correct interpretation for 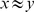 would be 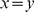 (i.e., the titles are identical). However, several issues made this test unrealistic. First, many titles are reported incompletely in search results (titles often end with “…” to indicate that the rest of the title has been omitted). Furthermore, we noticed the presence of significant numbers of mis-spellings in the titles of publications returned by search engines. We also observed discrepancies in the titles given by authors on their webpages and the titles reported online. We are unclear as to why these mis-spellings and discrepancies were so common; our approximation measure was an attempt to cope with these inconsistencies without overly penalizing our method.

### Different filter contributions

**Figure 2:**
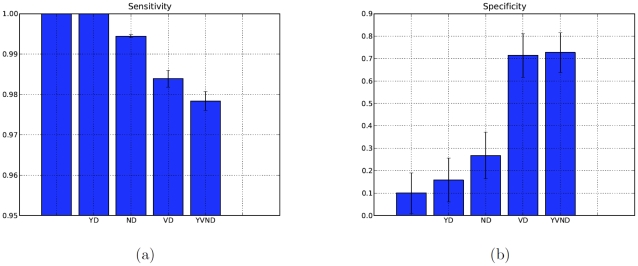
Performance of our method and individual filters. Our method achieves significant gains in specificity with little cost to sensitivity when all filters are used (Column *YNVD*) as compared to the raw results (left-most column). When considering the contribution of individual filters, vocabulary (Column *VD*) provides the greatest improvement while the year (Column *YD*) provides the smallest. Our more stringent name filter (Column *ND*) improves the accuracy somewhat, though the relatively small improvement confirms earlier assertions that name ambiguity is responsible for many of the incorrectly attributed publications.

We used our method to construct estimated publication-citation records for each of the individuals in the manually curated data set. In order to characterize both the performance of each filter as well as the overall system, we ran the method using the year, name, and vocabulary filters individually (but in combination with the duplication filter to eliminate double counting) and we also used the filters together as diagrammed in Figure 1. The average sensitivity and specificity scores for these different runs are shown in Figure 2. The first bar in either figure indicates the sensitivity/specificity when *no* filter is applied: this result is equivalent to taking the raw search engine results to be the estimate. The final bar depicts the performance of the method with all filters applied.

Since sensitivity measures the inclusion of true positives in the estimate, as more stringent filters are applied, the sensitivity suffers: filters only remove publications from the raw result returned by the search engine, so raw results will contain the most true positives. It is noteworthy that the sensitivity of these raw results are exactly or very near to 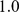, indicating that approximately all publications for each individual are being returned. The very low specificity score, however, (approximately 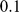) indicates that in addition to the correct publications, the search engine is returning many that have nothing to do with the individual of interest.

As filters are applied, we observe several trends:

#### Filters only slightly impact sensitivity

Though sensitivity drops monotonically, it does not decrease by a substantial amount. Even once all filters are in affect, the sensitivity is still around 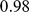, indicating that nearly all publications belonging to the individual are still in the estimate returned by our method.

#### The vocabulary filter significantly improves accuracy

The most substantial gain in accuracy is provided by the vocabulary filter. When beginning this project, we were aware that name ambiguity was a major source of error. As a result, it is not surprising that the vocabulary filter provides such dramatic increases in performance over the name and year filters. What is noteworthy is the extent to which the vocabulary filter improves the specificity—by approximately 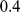.

#### Filters that improve specificity also degrade sensitivity

The year filter makes a very modest improvement to the specificity at no apparent cost to sensitivity, the name filter improves specificity somewhat more with a 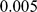 cost to sensitivity, and the vocabulary filter degrades sensitivity by approximately 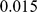 while boosting specificity by a dramatic 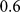. Thus, larger gains in specificity are correlated with larger (though still very minute) losses in sensitivity.

#### Specificity remains below 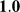


Specificity rises dramatically from 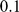 (no filters) to 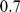 (all filters). This indicates that, while the estimates still include a number of publications which are not reported in the manually curated publication records, the number of extraneous publications is much smaller. In trying to understand this gap better, we manually inspected many of the results and discovered that many of the publications being classified as false positives are, in fact, authored by the individual of interest. In these cases, the mis-classified publications are either not mentioned by the author on her publications page (used to build the true publication record) or sufficiently mangled as to not satisfy our measure of approximate identity. Thus, it may be that for many individuals, our method has somewhat better specificity than is being reported here.

Overall, when filters are all applied (the final column), our method provides a factor of 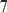 improvement in specificity with only a small cost to sensitivity. As we will discuss in later sections, these improvements translate into even greater improvements when citation indices are considered.

### Analysis of the vocabulary agreement filter

Though many individuals will provide complete publication lists on their webpages, it is common to encounter those that include only partial lists (either selected publications or lists that have not been recently updated). In either case, only some of the publication vocabulary will be available. How will such omissions affect the accuracy of our method? We consider both situations: where publications have been selected and where lists have not been recently updated.

#### Selected publications

**Figure 3:**
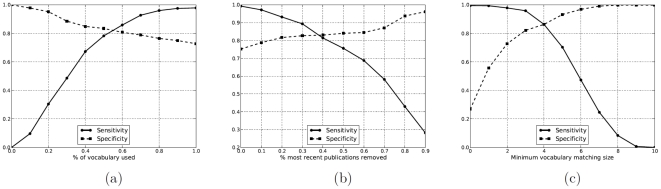
Moderate amounts of contextual information can significantly improve the accuracy of estimated citation records. We measured the impact of vocabulary on the sensitivity (solid line) and specificity (dashed line) of PCRs generated by our method. The amount of publication vocabulary used (a) shows that only about 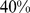 of publication vocabulary is required to obtain good estimates. Often publications lists provided by authors on the web will be unmaintained, in which case some recent publications will be missing. We considered this case (b) and found that even when 30% of recent publications are missing, estimates are still good. Finally, (c) shows how the number of those vocabulary words required to be found in a publication title (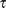) influences the accuracy of citation record estimates: while some vocabulary overlap is required to improve matches, an overlap of two to three is optimal in order to balance out improvements in specificity with degradation in sensitivity.

In order to study this circumstance, we re-ran our method on the curated data set, restricting our method to using only a percentage of the total vocabulary available on the publication list webpages. The vocabulary used was randomly selected. We assessed the sensitivity and specificity for a range of different vocabulary percentages. The results are shown in Figure 3(a). As expected, having little or no vocabulary produces terrible results (in the extreme, no publications are selected because the vocabulary set is empty). However, only 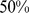 of the vocabulary data is required in order to achieve acceptable sensitivity/specificity scores (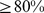). This suggests that, while the complete publications lists for an individual may not be readily available, a reasonable sample of the publications will still permit our method to reconstruct a good estimate of the individual's publication-citation record.

#### Out-of-date publication lists

In modeling this situation, we re-ran our method on the curated data set, using as filter vocabulary a subset of the publications in each individual's manually curated publication list. A percentage of the publications were removed, starting with those published most recently (e.g., in a publication record with 20 entries, 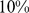 removal indicates that the 2 most recent entries were removed; 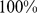 removal indicates that all entries were removed). We assessed the sensitivity and specificity for the full spectrum of unmaintained publication lists. The results are shown in Figure 3(b). As expected, using the full publication record produces the best results. As recent publications are removed, sensitivity falls. The curves indicate that, for the data set we considered, an author can omit upwards of 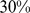 of their publication history without severely impacting the performance of our method.

Both of these results also suggests that, where publication lists are not available, a substantial body of text written by the individual about their research area might be an acceptable substitute. Given the wide variability of texts that can be considered summaries of research interests, we have not attempted to characterize the performance of our method on such data. However, this vocabulary source may give better results than taking the raw search results in instances where a publication list is not available.

We were also interested in understanding the effect of the vocabulary minimum matching size parameter, 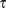, on the overall performance of the method. As described in the Methods section, this parameter determines how many of the vocabulary words must appear in a publication's title in order for the publication to be selected by the vocabulary filter. We ran our method on the curated data set for several values of 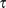, ranging from 0 to 10. The results are shown in Figure 3(c). In general, as the minimum matching size increases, specificity rises and sensitivity falls. Our method performs best in the range 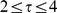. Depending upon one's relative interests in sensitivity (having all publications) and specificity (not including incorrect publications), different choices of 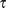 may be desirable. As we favored including publications of an individual, all results in this paper were generated using 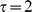.

### Impact of method estimates on publication indices

**Figure 4:**
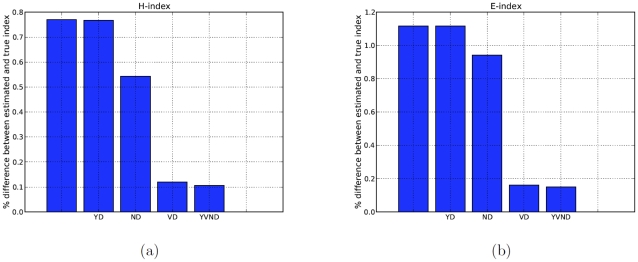
Citation records generated by our method's filters significantly reduces overestimates of h- and e-indices. (a) H-indices and (b) e-indices computed from raw citation records overestimate the true index values by approximately 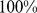. The filters introduced in our method (column *YVND*) reduce this overestimate by a factor of 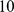.

Quite often publication-citation records are collected for the sole purpose of computing citation indices. From this vantage point, the accuracy of the estimated PCRs is less important than the accuracy of the indices computed from the estimated PCRs. In order to assess the accuracy of citation indices computed from the estimate PCRs generated by our method, we took our method's PCR estimates and computed h- and e-indices for each one. These values were then compared to the true h- and e-indices computed for the individuals in the manually curated data set (these could be computed precisely since we had the complete publication records for each individual). Figure 4 shows the average percent difference between the estimated indices and the true indices for several different combination of filters. When no filters are applied, the h- and e-indices are overestimated by 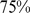 and 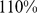, respectively. This overestimate has the effect of nearly doubling the true scores (an individual with an h-index of 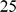 will have a reported score close to 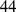). Once all filters are applied, this overestimate is reduced to 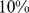 and 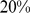, which in a normal range for h-index scores (between 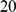 and 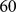), indicates that overestimates will be off by at most two to five points.

These results suggest that the reported specificity for our method, 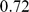, still generates highly accurate estimates of citation indices which, insofar as these are the objectives of PCR analysis, underscores the utility of this method.

### Performance comparison to existing approaches

All existing methods with which we are familiar that construct publication-citation records do not employ filters (note: the Web of Science advertises the capability to correctly select publications using only names, but in our studies we found no noticeable difference between Web of Science's results and those of any other publication search engine). As a result, the performance of these methods is comparable to the raw results returned by the online publication search engines. For the different definitions of accuracy considered (PCR and citation indices), these results are shown in Figures 2 and 4. On the curated data set we used, our method outperforms methods based on raw search results by between a factor of 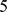 and 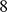, depending on whether actual publication-citation records or citation indices are considered. This difference in accuracy represents a significant improvement the quality of results being returned.

### Conclusions and future work

In this paper, we have presented a new method for the rapid and automated estimation of publication-citation records. In order to reconstruct the publication-citation record for an individual of interest, the method requires the individual's name as well as a webpage providing vocabulary that may be found in the individual's publication titles (usually this webpage will be a list of the individual's publications). Using a manually curated data set of publication-citation records, we have shown that our method reliably provides good estimates of the publication-citation records and the related citation indices. A fully-functional implementation of our method can be accessed at http://topp.ruthsresearch.org.

In the current academic environment, publication-citation records and citation indices are important for measuring individual and group performance. Computing these can often be a difficult, time-intensive, and error-prone process. We propose that our method can be used in two ways. First, often people performing these analyses do not have the time to carefully reconstruct citation records by hand. In these instances our method can provide estimates that can be used in place of the exact scores. Of course, some error is inevitable in these estimates, which motivates the second use case. In situations where time is available and absolute certainty of the records and indices are crucial, our method can be used as a first pass to identify a set of publications that can then be curated more carefully. Since many name queries can return hundreds or even thousands of results, using our method as a first pass can translate into many hours of saved time.

While our method does demonstrate high levels of accuracy, we recognize several areas for improvement. Since the vocabulary filter was the greatest source of improvement in specificity, but also the greatest loss in sensitivity, we consider that this filter should be the focus for future work. Misspellings, plurals, and other small modifications to words can have an impact on the recognition of vocabulary words in publication titles. Handling these and other cases could provide improvements in accuracy. Furthermore, we suspect that some improvement in accuracy may be gained by first clustering publications together that share similar vocabulary. These clusters could then be tested against the input vocabulary and either admitted or rejected together. This could possibly resolve issues with omitting publications that share no vocabulary with the input vocabulary, but have significant overlap with other publications that are flagged for inclusion.

Given the consequence of assessing individual, group, department, and university performance, we consider the question of reliable estimation of publication-citation records to be an important topic. In this paper, we have presented our method as a first approach that has both demonstrated the potential utility of such automated methods and identified productive directions for future work.

### Supplementary Information

The manually curated data set used to generate results are included in the supplementary material as Data Set S1. An implementation of our method is available at http://topp.ruthsresearch.org.

## Supporting Information

Data Set S1 Manually curated data.
(0.22 MB ZIP)
